# Simultaneous Detection of *Salmonella typhimurium* and *Escherichia coli O157:H7* in Drinking Water and Milk with Mach–Zehnder Interferometers Monolithically Integrated on Silicon Chips

**DOI:** 10.3390/bios12070507

**Published:** 2022-07-11

**Authors:** Michailia Angelopoulou, Panagiota Petrou, Konstantinos Misiakos, Ioannis Raptis, Sotirios Kakabakos

**Affiliations:** 1 Immunoassays–Immunosensors Lab, Institute of Nuclear & Radiological Sciences & Technology, Energy & Safety, NCSR “Demokritos”, 15341 Aghia Paraskevi, Greece; ypetrou@rrp.demokritos.gr; 2 Institute of Nanoscience & Nanotechnology, NCSR “Demokritos”, 15341 Aghia Paraskevi, Greece; k.misiakos@inn.demokritos.gr (K.M.); i.raptis@inn.demokritos.gr (I.R.)

**Keywords:** bacteria, *Salmonella typhimurium*, *Escherichia coli*, Mach–Zehnder Interferometry, immunosensor

## Abstract

The consumption of water and milk contaminated with bacteria can lead to foodborne disease outbreaks. For this reason, the development of rapid and sensitive analytical methods for bacteria detection is of primary importance for public health protection. Here, a miniaturized immunosensor based on broadband Mach–Zehnder Interferometry for the simultaneous determination of *S. typhimurium* and *E. coli O157:H7* in drinking water and milk is presented. For the assay, mixtures of bacteria solutions with anti-bacteria-specific antibodies were run over the chip, followed by solutions of biotinylated anti-species-specific antibody and streptavidin. The assay was fast (10 min for water, 15 min for milk), accurate, sensitive (LOD: 40 cfu/mL for *S. typhimurium*; 110 cfu/mL for *E. coli*) and reproducible. The analytical characteristics achieved combined with the small chip size make the proposed biosensor suitable for on-site bacteria determination in drinking water and milk samples.

## 1. Introduction

The consumption of food and water contaminated with pathogens is of global interest, as it leads to 48 million infections annually, resulting in 128,000 hospitalizations and 3000 deaths [1]. According to Centers for Disease Control and Prevention (CDC), the estimated incidents of foodborne illness caused by 31 pathogenic bacteria in the US amount to a total of 9 million cases per year, from which 20% is attributed to *Salmonella* spp., *Escherichia coli O157:H7*, *Staphylococcus aureus*, *Clostridium perfringens*, *Campylobacter* spp. and *Shigella* spp. [2]. Among them, *Salmonella typhimurium* (*S. typhimurium*) and *Escherichia coli O157:H7* (*E. coli O157:H7*) are both facultatively anaerobic, rod-shaped, gram-negative bacteria, belonging to the Enterobacteriaceae family. The ingestion of *S. typhimurium* causes fever, nausea, diarrhea, stomach discomfort, vomiting, dehydration and weakness, while *E. coli* may, in addition to the aforementioned symptoms, cause potentially life-threatening complications known as hemolytic uremic syndrome and hemorrhagic colitis. In both cases, the clinical symptoms may last from 5 to 7 days [3,4]. The high number of outbreaks due to *S. typhimurium* and *E. coli O157:H7* infections is also associated with high economic impact due to medical costs, loss of work hours and recalls of product suspicious for infection. The problem is intensified by the phenomenon of anti-microbial resistance (AMR), i.e., the limited effectiveness of antibiotics against many pathogens as a result of their overuse, which is expected to led in the near future to an increase in the number of deaths worldwide due to bacterial infections [5,6]. Thus, there is an urgent need for rapid methods for pathogen detection in order to minimize as much possible the bacteria outbreak effects in public health and the economy [7].

The conventional methods for bacteria detection and identification are based on culture and plating. Those methods are reliable but include several steps such as pre-enrichment, selective enrichment, isolation and confirmation through biochemical and serological tests, which are time-consuming since they require at least 5–7 days to complete. To shorten the analysis time to 2–4 days, ELISA- and DNA-based methods that do not require the selective plating steps have been employed for bacteria identification [8,9,10,11]. However, these methods are laboratory bound, require expensive instrumentation, and highly skilled personnel.

In recent years, biosensors based on electrochemical, piezoelectric or optical transducers are gaining ground in foodborne bacteria detection. Concerning electrochemical immunosensors, devices employing amperometric, potentiometric, impedemetric and conductimetric detection principles have been developed for the detection of bacteria [12,13,14,15]. Although these sensors claim inexpensive analysis and potential for miniaturization, they often require labels for signal enhancement to improve their detection limits. Immunosensors based on piezoelectric phenomena are capable for label-free detection of bacteria but they lack in sensitivity [16]. On the other hand, optical biosensors employing different transduction principles such as light absorbance, surface plasmon resonance (SPR), fluorescence, light polarization, and Raman scattering are powerful tools for foodborne bacteria detection [17]. Optical detection provides several advantages over other transduction principles such as less interference from the sample and ability for direct determination of pathogens in complex matrices with minimal sample treatment. Although SPR biosensors have been widely used for label-free bacteria detection, their limit of detection is usually higher than 10^3^ cfu/mL [18,19,20,21,22,23]. Among the label-free biosensors, interferometric ones are the most promising bacteria detection systems, as they offer high detection sensitivity, multiplexing capability and real-time determinations. Recently, a bi-modal interferometric sensor, an interferometric reflectance imaging sensor and a microcavity in-line Mach–Zehnder Interferometer have been employed for the detection of *E. coli* with detection limits of 40, 2.2 and 100 cfu/mL, respectively [24,25,26]. Moreover, another interferometric sensor based on white light reflectance spectroscopy has been developed for the detection of *S. typhimurium* in drinking water samples, exhibiting a detection limit of 320 cfu/mL [27].

**Figure 1:**
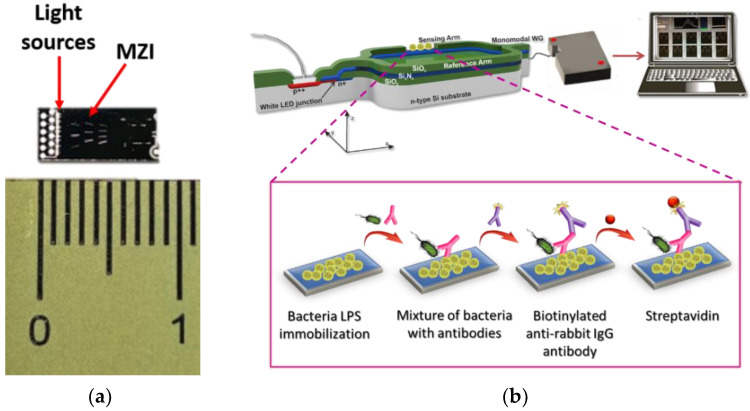
(**a**) Actual size of the chip and (**b**) 3D schematic of assay configuration for bacteria detection using the MZI sensor.

In this work, a label-free optical immunosensor based on arrays of Mach–Zehnder Interferometers (MZIs) monolithically integrated onto silicon chips is employed, for the first time, for the simultaneous detection of two bacteria, namely *S. typhimurium* and *E. coli* in water and milk samples. The silicon chip comprises 10 integrated MZIs along with their corresponding light sources (LEDs) that are silicon avalanche diodes emitting white light (Figure 1a and Figure S1). Thus, the MZI sensors employed in the current work are broadband integrated interferometers that exploit the broad spectrum of the integrated light source to resolve the two issues associated with the monochromatic MZI: signal fading and phase ambiguity [28]. The chip is covered by a silicon oxide cladding layer that has been selectively removed from a 600-μm long area over the sensing arm of each MZI to allow for interaction of the waveguided photons with the spotted onto the sensing arm biomolecules. The ten MZIs converge in a single output at the edge of the chip where the interference fringes over a broad spectral range are monitored by an external spectrometer. The spectral shifts are obtained by Fourier Transform (FT) and by keeping track of the phase at the FT maximum (our observable) [28]. This way the signal fading and phase ambiguity are eliminated and the noise is filtered out, while the extinction ratio makes no particular difference to the observable. The array of 10 multiplexed MZIs provides the opportunity to simultaneously monitor a multitude of different analytes (including non-specific binding) and obtain replicate determinations of the same analyte on a single chip. The MZI chips have been successfully utilized for the multiplexed label-free determination of allergens and mycotoxins in foodstuffs, as well as for the detection of goat milk and PDO cheeses’ adulteration with bovine milk [29,30,31,32]. The bacteria detection was performed employing a competitive immunoassay principle through the biofunctionalization of the sensing arm windows of different MZIs on the same chip with lipopolysaccharides (LPS) of the two bacteria (Figure 1b). The assay included reaction of the immobilized LPS molecules with a mixture of the sample with antibodies specific for the two bacteria, followed by reaction with biotinylated anti-species-specific antibodies and streptavidin. All reactions taking place on the LPS-modified sensing arm windows change the effective refractive index, causing a blue shift of the interference spectrum, which is transformed by Discrete Fourier Transform to a phase shift in radians. Several assay parameters have been optimized, aiming to fast and sensitive simultaneous determination of both bacteria in drinking water and milk.

## 2. Materials and Methods

### 2.1. Materials

*Salmonella enterica* serovar *typhimurium* (*S. typhimurium*, ATCC 14028), *Escherichia coli O157:H7* (*E. coli O157:H7*, NCTC 12900), *Escherichia coli* (*TOP10 Competent cells clone 2*, ATCC PTA-5689), and *Salmonella enterica* serovar *Thomson* were kindly provided from Delta Foods S.A. (Athens, Greece). Plate Count Agar (PCA) with skimmed milk was purchased from BIOKAR Diagnostics (Allonne, France). Petri dishes (92 mm, 16 mm), spreaders and polystyrene inoculation loops (1 μL), were obtained from Sarstedt AG & Co. KG (Numbrecht, Germany). Lipopolysaccharide (LPS) of *E. coli* was obtained from Creative Diagnostics (Shirley, NY, USA). The goat polyclonal antibody against *E. coli* was from Kirkegaard & Perry Lab Inc. (Gaithersburg, MD). The rabbit polyclonal antibody against *S. typhimurium*, donkey anti-goat IgG antibody and donkey anti-rabbit IgG antibody were purchased from Bio-Rad (Hercules, CA, USA). *Salmonella* LPS, bovine serum albumin (BSA), 3-aminopropyl-triethoxysilane (APTES) and buffered peptone water were purchased from Sigma-Aldrich (Darmstadt, Germany). Streptavidin and sulfosuccinimidyl-6-[biotin-amido]hexanoate) (EZ-Link™ Sulfo-NHS-LC-Biotin) was from Thermo-Scientific (Waltham, MA, USA). Full-fat highly pasteurized milk (DELTA, NOUNOU) and bottled water (ZAGORI, AVRA) were purchased from the local market. The water used in the study was doubly distilled. BioOdyssey™ Calligrapher MiniArrayer (Bio-Rad Laboratories Inc., Hercules, CA, USA) was used for the spotting of the chips with *S. typhimurium* and *E. coli* LPS solutions. Fabrication of the chips was performed as described previously [23,24]. The anti-species-specific antibodies were biotinylated according to a previously published protocol [33] as briefly described in the Supplementary Materials. The preparation of the LPS and bacteria calibrators is also described in the Supplementary Materials.

### 2.2. Sample Preparation and Pre-Enrichment

Bottled water and highly pasteurized cow milk were inoculated with *E. coli* and *S. typhimurium* at 1 × 10^7^ cfu/mL. These samples were then serially diluted with the respective uninoculated matrix in order to obtain the desired concentrations for both bacteria. To determine the least required pre-enrichment duration, 1 mL of 5 cfu/mL *S. typhimurium* or 7 cfu/mL *E. coli* suspension was spiked in 25 mL of highly pasteurized cow milk, stirred and transferred to a sterile bottle containing 225 mL peptone water. The mixtures were stirred and incubated for 16 h at 37 °C. In order to determine the bacteria proliferation rate, 1 mL of the above mixture was collected every 30 min for the first 8 h. From these samples, 100 μL appropriately diluted with sterile PBS were spread on PCA petri dishes, incubated overnight at 37 °C, and the number of colony-forming units was counted.

### 2.3. Chemical and Biological Functionalization of the Chip

For their chemical activation, the chips were cleaned and hydrophylized through O_2_ plasma treatment for 30 s, and immersed for 2 min in a 0.5% (*v*/*v*) APTES solution, rinsed with water, dried under nitrogen stream and heated at 120 °C for 20 min. The biological activation of the chips was performed using the BioOdyssey Calligrapher Mini Arrayer. In particular, as schematically depicted in Figure S1, 3 MZIs per chip were spotted with a 100 μg/mL *S. typhimurium* LPS solution, 4 MZIs with a 50 μg/mL *E. coli* LPS solution, and the remaining 3 with a 100 μg/mL BSA solution for the determination of non-specific binding. After spotting, the chips were placed in a humidity chamber overnight at 4 °C. Then, the biofunctionalized chips were washed, and incubated for 1 h in a 1% (*w*/*v*) BSA solution in 0.1 M NaHCO_3_, to block the non-specific binding sites on the sensing arm, rinsed with water and dried under the nitrogen stream.

### 2.4. Immunoassay for Bacteria Detection with MZI Immunosensor

The delivery of the samples over the chip surface was achieved through attachment of an appropriately designed microfluidic module [31]. Then, the chip was placed on a handling frame and inserted in the docking station of the measuring device. The reagents were flowed over the chip using a peristaltic pump. Prior to the assay, calibrators/samples were mixed with a solution of antibodies against *S. typhimurium* (0.75 μg/mL) and *E. coli* (0.5 μg/mL) at a 1:1 volume ratio, and incubated for 15 min. For the analysis of water samples, after chip equilibration with assay buffer, 100 μL of these mixtures were run over the chip at a rate of 35 μL/min, followed by 100 μL of a 10 μg/mL biotinylated anti-species-specific antibodies, and 100 μL of a streptavidin solution (Figure 1). The introduction of these solutions was achieved using an injector (Rheodyne 7725i) with a 100-μL loop. After assay completion, a regeneration step was followed in order to remove the bound antibodies and allow reuse of the chip. Thus, 100 μL of 0.05 M HCl solution and 100 μL of 0.05 M NaOH solution were pumped over the chip sequentially, followed by 100 μL of assay buffer for chip equilibration. For the determination of bacteria in milk samples, after the primary immunoreaction, 100 μL of washing solution were run (50 mM PBS, pH 7.4), followed by assay buffer prior to the introduction of biotinylated anti-species-specific antibodies. For the construction of the calibration curve and the determination of bacteria concentration in samples, the cumulative response of the MZIs during the reaction with the biotinylated anti-species-specific antibodies and streptavidin was considered after subtraction of the respective response from the MZIs spotted with BSA.

## 3. Results and Discussion

### 3.1. Optimization of Assay Conditions

The detection of bacteria in drinking water and milk samples was based on a competitive immunoassay between the immobilized onto the MZIs LPS and the bacteria in the sample for binding to the specific antibody. Then, reaction with biotinylated anti-species-specific antibodies and streptavidin were applied for signal enhancement (three-step assay), aiming to shorten as possible the assay duration. Several parameters including the concentration of the LPS solution used for coating, the concentration of the bacteria-specific antibodies and the reagents flow rate have been optimized to achieve the maximum signal in the absence of bacteria and the highest percent signal drop in the presence of bacteria calibrators. The relative results are presented in the Supplementary Materials. Thus, the LPS concentration used for spotting was 50 μg/mL and 100 μg/mL for *S. typhimurium* and *E. coli* LPS (Figure S2) in combination with 0.75 and 0.5 μg/mL of antibody against *S. typhimurium* and *E. coli* (Figure S3), respectively. The specificity of anti *S*. *typhimurium* and anti *E. coli* antibodies against each other was assessed using chips with MZIs spotted with *E. coli* or *S. typhimurium* LPS and running the zero calibrator of each pathogen (containing one anti-bacterium antibody) at a time followed by the respective biotinylated anti-species-specific antibody and streptavidin. It was found that signal was obtained only from the MZIs spotted with the LPS of the pathogen for which the zero calibrator was run, whereas no detectable response was observed from the MZIs coated with the LPS of the other bacteria or with BSA. The reagents flow rate that also determines the assay duration, since a fixed volume loop was used for the introduction of different solutions, was also optimized. As a compromise between short assay duration, high absolute signal and detection sensitivity (Figure S4), the flow rate of 35 μL/min was selected for the final protocol, leading to an assay duration of only 10 min.

### 3.2. Effect of Pre-Incubation

**Figure 2:**
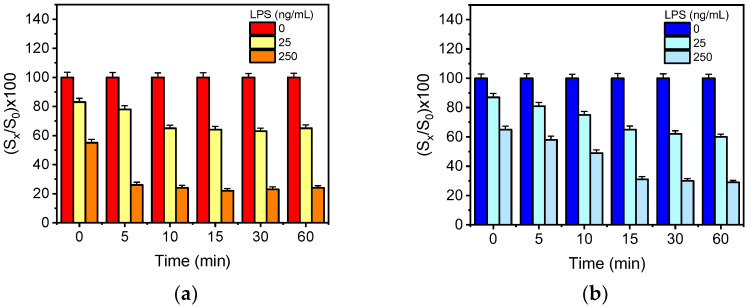
(**a**) Percent signal values obtained for a calibrator containing 25 (yellow columns) or 250 ng/mL of *E. coli* LPS (orange columns) with respect to zero calibrator (red columns) without pre-incubation and with 5-, 10-, 15-, 30- and 60-min pre-incubation of the calibrators with anti-*E. coli* antibody solution. (**b**) Percent signal values obtained for a calibrator containing 25 (cyan columns) or 250 ng/mL of *S. typhimurium* LPS (light blue columns) with respect to zero calibrator (blue columns) without pre-incubation and with 5-, 10-, 15-, 30- and 60-min pre-incubation of the calibrators with anti-*S. typhimurium* antibody solution. Each point is the mean value of three measurements. Error bars correspond to ±SD.

The detection sensitivity of a competitive assay, i.e., the percent signal drop observed in the presence of the analyte with respect to the zero calibrator signal, could be significantly improved through pre-incubation of the calibrators/samples with the antibody prior to the reaction with the immobilized antigen. To investigate the effect of pre-incubation in the detection sensitivity, mixtures of LPS calibrators of both bacteria (25 and 250 ng/mL) with the respective specific anti-bacteria antibodies were incubated for 5, 10, 15, 30 and 60 min and then run over the chips. The signals obtained from the assay with the pre-incubated mixtures were compared to those provided without pre-incubation. As it is presented in Figure 2a, the percent signal obtained for the *E. coli* LPS calibrator with a concentration of 25 ng/mL with respect to the zero calibrator dropped from 83% to 78% after 5 min of pre-incubation, whereas the signal of the calibrator with a concentration of 250 ng/mL dropped from 55% to 26%. Moreover, when increasing the pre-incubation time from 5 to 10 min, the signal obtained for the calibrator with the lower concentration dropped further to 65%, whereas the respective signal drop for the calibrator with the higher concentration did not alter. An increase of pre-incubation time from 10 up to 60 min did not further improve the detection sensitivity, compared to 10 min. Regarding *S. typhimurium* (Figure 2b), the percent signal obtained for the calibrators with concentrations 25 and 250 ng/mL, after 15 min of pre-incubation, dropped from 87% to 65% and from 65% to 30%, respectively. Longer pre-incubation times provided similar signal drops to those obtained after 15 min of pre-incubation for both calibrators. Based on these results and aiming to the simultaneous determination of *E. coli* and *S. typhimurium*, a 15 min pre-incubation of calibrators/samples with the specific antibodies was adopted to the final protocol.

### 3.3. Matrix Effect of Drinking Water and Milk

**Figure 3:**
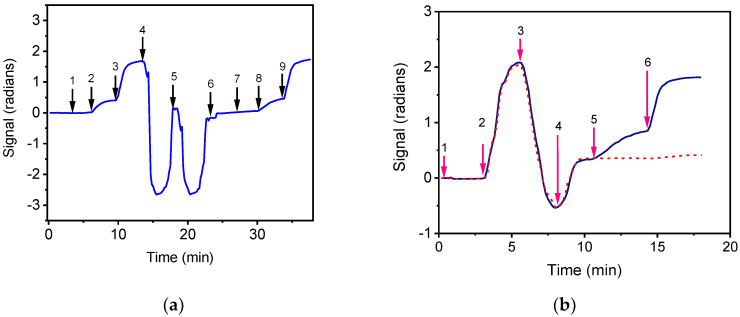
(**a**) Real-time response obtained for *S. typhimurium* zero calibrator prepared in assay buffer (arrow 1 to 4) or in bottled water (arrow 7 to end). The arrows indicate the sequence of solutions passing over the chip: assay buffer (start to 1); zero calibrator in assay buffer (arrow 1 to 2); biotinylated anti-species specific antibody (arrow 2 to 3); streptavidin (arrow 3 to 4); 50 mM HCl (arrow 4 to 5); 50 mM NaOH (arrow 5 to 6); assay buffer (arrow 6 to 7); zero calibrator in bottled water (arrow 7 to 8); biotinylated anti-species specific antibody (arrow 8 to 9); streptavidin (arrow 9 to end). (**b**) Real-time response obtained for *E. coli* zero calibrator prepared in milk. The arrows indicate the sequence of solutions passing over the chip: assay buffer (arrow 1 to 2), zero calibrator prepared in milk (arrow 2 to 3), washing buffer (arrow 3 to 4), assay buffer (arrow 4 to 5), biotinylated anti-species-specific antibody (arrow 5 to 6) and streptavidin (arrow 6 to end). Dashed line corresponds to non-specific binding signal.

The effect of bottled water on both the zero calibrator signals of *E. coli* and *S. typhimurium* and the detection sensitivity of the respective assays was investigated. For this purpose, *E. coli* and *S. typhimurium* zero calibrators were prepared in assay buffer as well as in bottled water. As it is shown in Figure 3a indicatively for *S. typhimurium*, the signal obtained from zero calibrator prepared in bottled water was similar to that of the zero calibrator prepared in assay buffer. Moreover, the calibration curves obtained with calibrators prepared in both matrices were almost identical.

Milk is an opaque liquid containing fats, proteins and minerals, which could influence assay performance. To investigate the effect of milk, commercial highly pasteurized full-fat cow milk, which did not contain detectable concentrations of bacteria, was used for the preparation of calibrators. As shown in Figure 3b indicatively for *E. coli*, the sensor response was affected by the presence of milk probably due to scattering of waveguided light and to non-specific binding of milk components on the sensor surface. To minimize such an effect, washing buffer and assay buffer were flowed over the chip after the first assay step, in order to wash out the milk and enable the monitoring of the sensors’ response due to the reaction of biotinylated antibodies and streptavidin (second and third assay steps). It was found that by implementing these washing steps, the signals obtained for *E. coli* and *S. typhimurium* zero calibrators prepared in milk were almost equal with those of calibrators prepared in assay buffer. In addition, the calibration curves obtained with calibrators prepared in either milk or assay buffer were superimposed. Thus, the calibrators for both bacteria were prepared in assay buffer and washing after the first assay step was implemented when milk samples were analyzed, increasing the total assay time to 15 min. It should be noticed that the waveguides spotted with BSA did not provided any detectable signal upon reaction with biotinylated anti-species-specific antibodies and streptavidin as shown in Figure 3b for the calibrator prepared in milk.

### 3.4. Effect of Bacteria Lysis to Assay Detection Sensitivity

**Figure 4:**
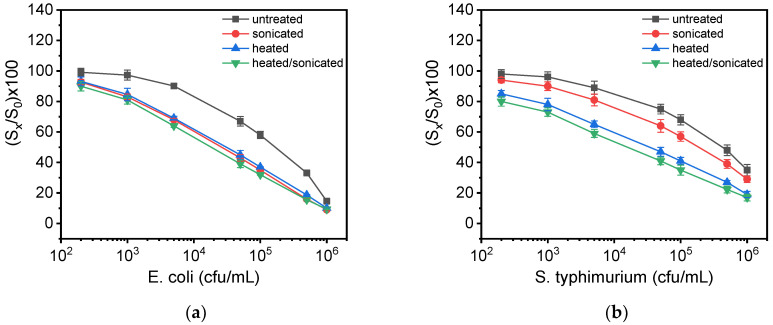
Calibration curves obtained for (**a**) *E. coli* and (**b**) *S. typhimurium* with calibrators prepared using live untreated (grey squares), heat-treated (blue triangles), ultra-sonicated (red circles) or heat-treated and ultra-sonicated bacteria (green triangles). Each point is the mean value of three measurements. Error bars correspond to ±SD.

In the literature, it is mentioned that the treatment of samples containing bacteria with detergent, heat/osmotic shock or sonication to achieve their lysis could noticeably improve the detection sensitivity of immunoassays [34]. For this reason, 1 mL of suspension containing *S. typhimurium* or *E. coli* at a concentration of 10^7^ cfu/mL were treated as follows: (a) heated at 90 °C for 10 min, (b) ultra-sonicated at 65% power for 5 min with 30 s intervals, by using an ultra-sonic disruptor, while the sample was immersed in an ice bath, and (c) heated and ultra-sonicated for 10 and 5 min, respectively. The treated suspensions were then used for the preparation of bacteria calibrators and the calibration curves obtained were compared with that obtained using calibrators prepared with untreated live bacteria. As it is shown in Figure 4a, both ultra-sonication and heat-treatment as well as their combination improved the detection sensitivity of *E. coli* by 15 times compared to live untreated bacteria. In the case of *S. typhimurium* (Figure 4b), the detection sensitivity was improved 9 times after ultra-sonication and up to 50 times after thermal treatment; the highest detection sensitivity was achieved when combining heat treatment with ultra-sonication (100 times improvement). Based on these results, for the simultaneous determination of *E. coli* and *S. typhimurium* in drinking water and milk samples, the thermal treatment at 90 °C for 10 min followed by 5 min ultra-sonication was selected. Characteristic real-time responses obtained from a chip on which different MZIs have been spotted with the LPS of *E. coli* (4 MZIs) or *S. typhimurium* (3 MZIs) when passing calibrators containing 1 × 10^3^ to 1 × 10^6^ cfu/mL bacteria, which have been subjected to heat-treatment and ultra-sonication, are presented in Figure S5.

### 3.5. Analytical Characteristics and Calibration Curves Using the MZI Chip

**Figure 5:**
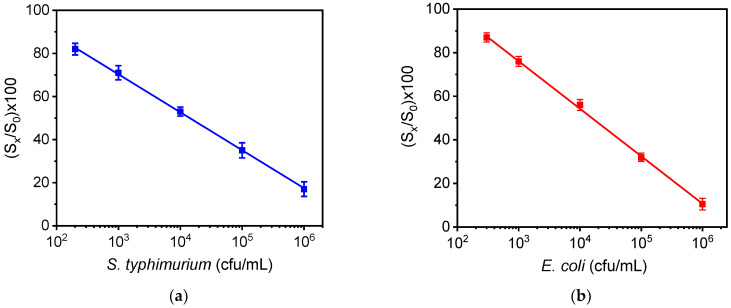
Calibration curves of (**a**) *S. typhimurium* and (**b**) *E. coli*. (S_x_/S_0_) × 100 represents the percent ratio of each calibrator signal (S_x_) to the zero calibrator signal (S_0_). Each point is the mean value of three chips ±SD.

In Figure 5a,b, the calibration curves for *S. typhimurium* and *E. coli* are provided, respectively. The dynamic range of *S. typhimurium* and *E. coli* assays ranged from 2 × 10^2^ to 1 × 10^6^ and from 3 × 10^2^ to 1 × 10^6^, respectively. The limit of detection of the assays was determined as the concentration corresponding to the signal equal to −3SD of the mean zero calibrator signals (28 replicate values for *E. coli* and 21 replicate values for *S*. *typhimurium* from 7 chips) and was 40 cfu/mL and 110 cfu/mL for *S. typhimurium* and *E*. *coli*, respectively.

The accuracy of the assay was also determined through recovery experiments. Thus, samples of bottled water and full-fat highly pasteurized cow milk that did not contain detectable levels of bacteria were spiked with bacteria at three different concentration levels (5 × 10^3^, 5 × 10^4^ and 5 × 10^5^ cfu/mL) and were analyzed in triplicate. The % recovery was calculated according to the equation:

(Bacteria concentration determined/Bacteria concentration added) × 100

As shown in Table S1, the recovery values ranged from 90 to 112%, indicating the high accuracy of the assay performed using the MZI chip. The repeatability of the assay was determined using bottled water and milk samples spiked with four different concentrations of the bacteria. The intra-assay coefficients of variation (CVs) were calculated after repetitive measurements of the bottled water and milk samples during the same day, whereas the inter-assay CVs were determined by measuring the samples in seven different days in a period of one month and were less than 5% and 7%, respectively.

Furthermore, cross reactivity experiments were performed for each assay, using in addition to *S. typhimurium* and *E. coli O157:H7*, *S*. *Thomson* and *E. coli top10*, both selected due to genetic similarities to targeted bacteria. Thus, calibrators containing 5 × 10^3^–5 × 10^7^ cfu/mL of *S. typhimurium*, *E. coliO157:H7*, *E. coli top10* and S. *Thomson,* and run in chips spotted either with *S. typhimurium* or *E. coli* LPS and the cross-reactivity, was evaluated through the equation:

%CR = (IC_50_ of target pathogen/IC_50_ of cross-reactant pathogen) × 100

**Figure 6:**
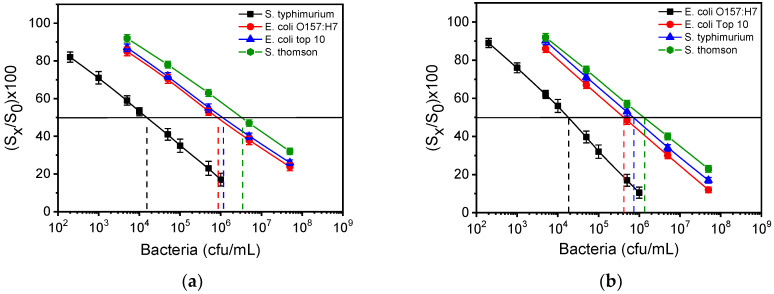
(**a**) Calibration curves for *S. typhimurium* (black squares), *E. coli O157:H7* (red circles), *E. coli top10* (blue triangles) and *S*. *Thomson* (green hexagones) obtained from MZIs spotted with *S. typhimurium* LPS. (**b**) Calibration curves for *E. coli O157:H7* (black squares), *E. coli top10* (red circles), *S. typhimurium* (blue triangles) and S. *Thomson* (green hexagones) obtained from MZIs spotted with *E. coli* LPS. The dashed vertical lines indicate the bacteria concentration that corresponds to a 50% signal drop with respect to zero calibrator (horizontal black line). Each point is the mean value of seven waveguides per chip ± SD.

The IC_50_ value is determined as the bacteria concentration that corresponds to a 50% signal drop with respect to the zero calibrator. As shown in Figure 6a, the cross reactivity values for the *S. typhimurium* assay were 1.7% for *E. coli O157:H7*, 1.2% for *E. coli top10*, and 0.4% for *S*. *Thomson*. In case of *E. coli O157:H7* (Figure 6b), the cross-reactivity values determined were 4.3%, 2.4%, and 1.3% for *E. coli top10*, *S. typhimurium* and *S*. *Thomson,* respectively. These results indicate the high specificity of antibodies against *S. typhimurium* and *E. coli O157:H7*.

### 3.6. Regeneration and Stability of the Immunosensor

Chip regeneration could reduce the total analysis cost by reusing the same chip for consecutive measurements. Thus, different regeneration solutions, i.e., 50 mM HCl, 50 mM NaOH, and a commercially available IgG elution buffer were tested to determine the one that could efficiently remove all bound molecules without affecting the immobilized onto the MZIs biomolecules. More specifically, 100 μL of each regeneration solution was flown over the chip after running the bacteria zero calibrators. Then, biotinylated anti-species-specific antibodies and streptavidin was run over the chip to determine the efficiency of bound molecules removal. It was found that the chip could be completely regenerated by flowing sequentially 50 mM HCl for 180 s, followed by 50 mM NaOH for another 180 s. Using the selected regeneration procedure, the reusability of the chip was also evaluated through repetitive assay/regeneration cycles. As shown in Figure S6, the zero calibrator signals obtained from chips functionalized with LPS of *S. typhimurium* were stable after 20 regenerations, whereas the signals obtained with chips spotted with LPS of *E. coli* started to decline after 12 assay/regeneration cycles, setting the limit of possible regenerations for the simultaneous determination of the two bacteria.

The stability of the LPS spotted chips was determined using chips stored dry in a desiccator at 4 °C at different time intervals over a period of one month. It was found that the responses obtained from these chips were not statistically different from those received from freshly prepared ones.

### 3.7. Sample Enrichment for Single Bacteria Detection

**Figure 7:**
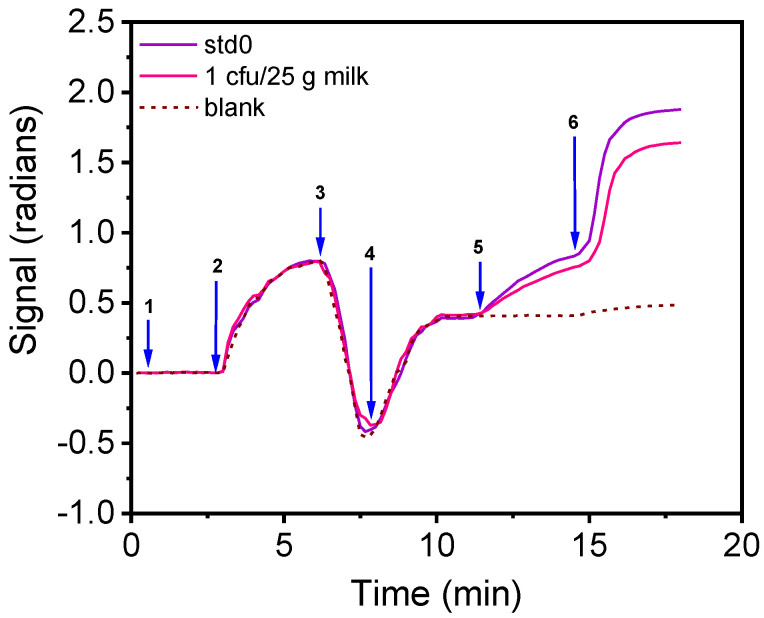
Real-time immunosensor responses when running a zero calibrator over the chip prepared in 10% milk in peptone water (purple line) and a milk sample initially containing 1 cfu of *E. coli*/25 g milk (pink line) after 7.5 h pre-enrichment. The arrows indicate the sequence of solutions passing over the chip: assay buffer (arrow 1 to 2), zero calibrator/sample in 10% milk in peptone water (arrow 2 to 3), washing (arrow 3 to 4), assay buffer (arrow 4 to 5), biotinylated anti-species-specific antibody (arrow 5 to 6) and streptavidin (arrow 6 to end). Dashed line corresponds to non-specific binding signal.

According to EU legislation, *Salmonella* spp. and *E. coli* should not be detected (zero tolerance) in 25 g of food (CE 1441/2007), including milk and drinking water. Thus, a pre-enrichment of the samples is required for the detection of the two bacteria with the proposed immunosensor. To determine the least needed pre-enrichment step duration for milk samples, 25 mL of full-fat pasteurized cow milk were spiked with 1 mL containing 7 cfu/mL for *E. coli* or 5 cfu/mL of *S. typhimurium*, mixed with 225 mL peptone water, and incubated at 37 °C. From these mixtures, 1-mL samples were collected every 0.5 h and their bacteria concentration was determined by plating and counting. It was found that after 7.5 h of enrichment, *E. coli* concentration reached 1625 cfu/mL, whereas the concentration of *S. typhimurium* reached 1107 cfu/mL. Thus, by adopting a pre-enrichment step of 7.5 h, it is expected that the immunosensor developed could detect a single bacteria in the milk. To confirm this, in Figure 7, the real-time signal provided for the zero calibrator and a milk sample containing initially 1 cfu/25 mL after 7.5 h of pre-enrichment is presented. As shown, the signal drop obtained from the milk sample with respect to the zero calibrator signal corresponds to approximately 300 cfu/mL. Similar results were obtained for water samples.

### 3.8. Comparison with Other Optical Label-Free Immunosensors

In Table 1, a comparison of the proposed MZI immunosensor with other label-free optical immunosensors, reported in literature, for the detection of *S. typhimurium* and *E. coli O157:H7* in terms of LOD, assay time and the type of the sample used, is presented. SPR immunosensors represent the most abundant category of label-free optical biosensors applied to the detection of pathogenic bacteria in food. In terms of *E. coli* detection, the proposed MZI immunosensor provided comparable LODs with those obtained for the detection of this pathogen in water and orange juice, as well as in milk, apple juice and ground beef extracts, with an SPR sensor [35] and a fiber optic SPR sensor [36]. An ultra-sensitive SPR biosensor for the detection of *E. coli* after immunomagnetic separation with an LOD of 3 cfu/mL in the assay buffer and analysis duration less than 70 min has also been reported [37]. The method has been applied in water samples (lake water and tap water); however, the LODs were not referred [37]. Wang et al. demonstrated an inhibition assay for the detection of *E. coli* using an SPR biosensor. The sensor provided an LOD of 3 × 10^4^ cfu/mL in a total assay time of 2 h, excluding sample pre-incubation and centrifugation [19]. Furthermore, an Interferometric Reflectance Imaging sensor (IRIS) was capable of detecting *E. coli* at a concentration as low as 2 cfu/mL in tap water. However, the assay duration was approximately 12 times longer (2 h) than that of the proposed immunosensor [26].

For the *S. typhimurium* detection, the majority of the SPR sensors provided LODs >10^5^ cfu/mL [20,21,22,23,38,39]. Only two biosensors, one based on SPR [40] and another on optical grating coupling [41] with LODs at the order of 10^3^ have been reported, the analysis duration of which was 20 and 60 min, respectively [40,41]. An Ω-shaped fiber optic LSPR sensor provided a three times higher LOD (128 cfu/mL) than that obtained with the MZI immunosensor, with an assay duration of 100 min [42], while a fiber optic biosensor was capable of detecting *S. typhimurium* in milk in less than 20 min with, however, a LOD 6 times higher (247 cfu/mL) than that achieved with the proposed immunosensor [43]. In another report, an SPR sensor could detect 7 cfu/mL of *S*. *typhimurium* in romaine lettuce in less than 6 min after 24 h pre-enrichment [44]. On the other hand, the proposed MZI sensor could detect 1 cfu/mL following a 7.5 h enrichment step before the analysis, which is completed in 10 min. An immunosensor based on Hartman interferometry exhibited a 250 times higher LOD than that obtained with the MZI sensor developed for a similar assay duration [45]. Finally, in a previous publication, our group has demonstrated an immunosensor based on White Light Reflectance Spectroscopy (WLRS) for the detection of *S. typhimurium* in drinking water samples with an eight times higher LOD and five times longer assay duration compared to the proposed MZI sensor [28].

In addition to immunosensors targeting the detection of a single pathogen, a few reports regarding the simultaneous detection of *Salmonella* spp. and *E. coli* have been also published. Bougelia et al. developed a fluidic-less device for the label-free “Culture-Capture-Measure” of *S*. *enteritidis*, *E. coli O157:H7* and *Streptococcus pneumoniae* through a microarray coupled to an SPR imager. This sensor was able to detect initial bacteria concentration as low as 2.8 cfu/mL and to monitor their proliferation in real-time. This was achieved through the placement of a growth chamber, on which the temperature was adjusted at 37 °C, on the SPR sensing surface. Nevertheless, to achieve this very low LOD the assay duration was extended up to 10 h [46]. Furthermore, a multichannel SPR sensor could detect 4 bacteria in apple juice with 30 times higher LOD and 3 times longer assay duration than that obtained with the MZI sensor regarding *E. coli* [34]. Another SPR biosensor has been reported for the detection of *Escherichia coli O157:H7*, *S. enteritidis* and *Listeria monocytogenes*. In comparison with this sensor, the proposed immunosensor provided 600 times higher sensitivity in terms of *E. coli* determination [47]. Vassocherova-Lisalova et al. developed an SPR method for the simultaneous detection of *E. coli* and *Salmonella* spp. in complex matrices, such as cucumber and hamburger. The LODs obtained for *E. coli* were approximately 2–6 times lower for cucumber and hamburger, respectively, whereas the LODs provided for *Salmonella* were 200–300 times higher, than those obtained with the MZI sensor [48]. Furthermore, the assay time was 8-fold longer. Waswa et al. could also detect *S. enteritidis* and *E. coli O157:H7* in a single run with an SPR sensor. The LOD achieved for *E. coli* in milk was four times lower than that obtained with the proposed MZI sensor, whereas the assay duration was two times longer [4]. The simultaneous detection of *Escherichia coli O157:H7*, *S*. *enteritidis* and *Listeria monocytogenes* in ready-to-eat food using a fiber optic immunosensor has been also demonstrated. In terms of *E. coli* detection, this sensor could detect 100 times higher concentrations than the MZI sensor developed applying, however, a 24 h pre-enrichment and a 4 h assay [49]. Overall, the proposed immunosensor is among the most sensitive and fast immunosensors reported for the detection of *S. typhimurium* and *E. coli O157:H7*. Employing a 10 min heat-treatment, 5 min ultra-sonication and 15 min pre-incubation of the sample with the antibody, 40 cfu/mL of *S. typhimurium* and 110 cfu/mL of *E. coli* could be detected with the proposed sensor within 10 min. In addition, by implementing a 7.5 h pre-enrichment step, 1 cfu per 25 g of food could be detected, thus meeting the limits set by the European Food Safety Authority guidelines.

**Table 1:** Comparison of the proposed MZI immunosensor with other optical label-free immunosensors.

Immunosensor	Target Bacteria	Sample	LOD (cfu/mL)	Assay Time (min)	Ref.
Fiber optic SPR	*E. coli O157:H7*	Water Orange juice	94	15	[35]
SPR	*E. coli O157:H7*	Milk Apple juice Ground beef extracts	10^2^–10^3^	30	[36]
SPR	*E. coli O157:H7* (immunomagnetic separation)	Buffer Water (lake, tap)	3 -	<70	[37]
SPR	*E. coli O157:H7*	Buffer	3 × 10^4^	120	[19]
IRIS sensor	*E. coli O157:H7*	Tap water	2.2	120	[26]
Lab-built SPR	*S. typhimurium*	Buffer	10^6^	6–7	[20]
SPR	*S. typhimurium*	Buffer	10^5^	10	[38]
SPR	*S. typhimurium*, *S. enteritidis*	Milk	2.5 × 10^5^ 2.5 × 10^8^	~100	[39]
SPR	*S. typhimurium*	Chicken carcass	1 × 10^6^	~17	[21]
SPR Imaging	*S. enteritidis*	Buffer Chicken carcass rinse	2.1 × 10^6^ 7.6 × 10^6^	20	[22]
Portable SPR	*S. typhimurium*.	Buffer	10^7^	~60	[23]
SPR	*Salmonella* group B, D, E	Buffer	1.7 × 10^3^	22	[40]
Fiber optic	*S. typhimurium*	Milk	247	<20	[43]
Optical grating coupler	*S. typhimurium*	Buffer	1.3 × 10^3^	60	[41]
SPR	*S. typhimurium*	Buffer	10^6^	≤120	[24]
SPR	*S. typhimurium*	Romaine lettuce	10^6^ 7	<6 24 h pre-enrichment	[44]
Ω-shaped fiber optic LSPR	*S. typhimurium*	Buffer	<128	100	[42]
Hartman Interferometry	*S. typhimurium*	Buffer Chicken carcass	10^4^	10	[45]
WLRS	*S. typhimurium*	Tap and bottled water	320	15	[28]
SPR	*Escherichia coli O157:H7* *S. enteritidis*, *Listeria monocytogenes*	Buffer	0.6 × 10^6^ 1.8 × 10^6^ 0.7 ×10^7^	5	[47]
Fiber optic	*Listeria monocytogenes Escherichia coli O157:H7* *S. enteritidis*	Ready-to-eat food (ground beef, chicken, turkey)	10^3^	240 (+24 h pre-enrichment)	[49]
SPR imaging	*E. coli O157:H7* *S. enteritidis* *Streptococcus pneumoniae*	Buffer	2.8	>6000	[46]
SPR	*Escherichia coli O157:H7* *Salmonella* spp.	Cucumber/ hamburger	57/17 7.4 × 10^3^/11.7 × 10^3^	<80	[48]
multi-channel SPR	*Escherichia coli O157:H7* *S. choleraesuis*, *Listeria monocytogenes*, *Campylobacter jejuni*	Apple juice	3.4 × 10^3^–1.2 × 10^5^	30	[34]
SPR	*Escherichia coli O157:H7* *Salmonella enteritidis*	Milk	25 23	32	[4]
MZI developed immunosensor	*Escherichia coli O157:H7* *S. typhimurium*	Bottled water/ Milk	110 40 1 cfu/mL after 7.5 h pre-enrichment	10/15	

## 4. Conclusions

The simultaneous determination of *S. typhimurium* and *E. coli* in drinking water and milk samples by an immunosensor based on arrays of MZIs integrated onto silicon chips was presented for the first time. The sensor provided real-time detection of the two bacteria in bottled water within 10 min, employing a three-step assay configuration, whereas the detection in milk was completed in 15 min. The assay was accurate, repeatable and sensitive with detection limits at the order of ≤10^2^ cfu/mL. The detection sensitivity was improved by approximately 15 and 100 times for *E. coli* and *S. typhimurium*, respectively, when the sample was heat-treated and sonicated prior to the analysis. Based on the detection sensitivity of the proposed sensor and taking into account that according to EU legislation there is zero tolerance for both *S*. *typhimurium* and *E. coli* in milk and water samples, the sample pre-enrichment required for a single bacteria detection was determined to be 7.5 h. Thus, the sample pre-enrichment and analysis can be completed in a single working day, suppressing considerably the sampling to analysis time required. Therefore, it is expected that the proposed sensor could find wide application in a Drinking Water Distribution System as well as in dairy industries for fast bacteria detection.

## Data Availability

The data presented in this study are available on request from the corresponding author.

## Supplementary Materials

The following supporting information can be downloaded at: https://www.mdpi.com/article/10.3390/bios12070507/s1, Figure S1: Depiction of chip-spotting layout; Figure S2: Optimization of LPS concentration used for coating; Figure S3: Optimization of anti-bacteria specific antibody concentration; Figure S4: Optimization of flow rate; Figure S5: Real-time responses from a chip spotted with the LPS of *E. coli* and *S. typhimurium*; Table S1: Results of recovery experiment; Figure S6: Regeneration of the immunosensor.
