# Supplementary material for: Simultaneous Detection of Salmonella typhimurium and Escherichia coli O157:H7 in Drinking Water and Milk with Mach–Zehnder Interferometers Monolithically Integrated on Silicon Chips
Source: Biosensors (Basel). 2022 Jul 11;12(7):507. doi: 10.3390/bios12070507 (PMC9313075; doi:10.3390/bios12070507)
Supplement: Supplementary file 1 [file biosensors-12-00507-s001.zip › biosensors-1774750-supplementary.pdf]

Supplementary material

Simultaneous Detection of Salmonella *typhimurium* and Escherichia *coli O157:H7* in Drinking Water and Milk with Mach–Zehnder Interferometers Monolithically Integrated on Silicon Chips

Michailia Angelopoulou [1,*], Panagiota Petrou [1], Konstantinos Misiakos [2], Ioannis Raptis [2] and Sotirios Kakabakos [1*]

[1] Immunoassays–Immunosensors Lab, Institute of Nuclear & Radiological Sciences & Technology, Energy & Safety, NCSR "Demokritos", Aghia Paraskevi 15341, Greece; ypetrou@rrp.demokritos.gr (P.P.)

[2] Institute of Nanoscience & Nanotechnology, NCSR "Demokritos", Aghia Paraskevi 15341, Greece; k.misiakos@inn.demokritos.gr (K.M.); i.raptis@inn.demokritos.gr (I.R.)

* Correspondence: mikangel@ipta.demokritos.gr (M.A.); skakab@rrp.demokritos.gr (S.K.); Tel.: +30-2106503819 (M.A. & S.K.)

1. Biotinylation of Anti-Species-Specific Antibodies

For the biotinylation, the concentrations of donkey anti-rabbit IgG and donkey anti-goat IgG antibodies were adjusted to 1 mg/mL with 0.25 M carbonate buffer, pH 9.1, containing 0.9% (w/v) NaCl. Then, a 100 mg/mL solution of sulfo-NHS-LC-biotin in DMSO was mixed with the antibody solution for the weight ratio of sulfo-NHS-LC-biotin to antibody to be 1.5:1, and left to react for 2 h, at RT. Then, the reaction mixtures were dialyzed against 0.1 M $NaHCO_3$ solution, pH 8.5, 0.9% (w/v) NaCl, 0.05% (w/v) NaN_3.

2. Bacteria Culturing and Counting

Bacteria strains (S. typhimurium, S. Thomson, E.coli O157:H7 and E. coli top10) were cultured on PCA petri dishes overnight at 37 °C. Then, a number of bacteria colonies was collected and suspended in 1 mL sterile phosphate buffer saline (PBS) 10 mM, pH 7.4. These suspensions were serially diluted and the viable bacteria concentration was determined by plate counting. For this purpose, 100 μL of each bacteria dilution were inoculated on PCA petri dishes and incubated overnight at 37 °C. The number of bacterial colonies formed on PCA were counted and expressed as colony forming units per milliliter (cfu/mL).

3. Preparation of calibrators

S. typhimurium LPS was reconstituted in carbonate buffer, pH 9.2 (coating buffer) at a concentration of 1 mg/mL, aliquoted and stored at −20 °C until use. Lyophilized E. coli O157:H7 LPS was diluted in distilled water at a concentration of 1 mg/mL, heated to about 50 °C with intermittent vortexing, and left for 1 h at RT to achieve a homogeneous solution, which was stored at 4 °C. The LPS stock solutions were used for the preparation of calibrators in 50 mM PBS buffer, pH 7.4, containing 10 g/L BSA (assay buffer). To prepare bacteria calibrators, suspensions of 1×10^8 cfu/mL in 50 mM PBS, pH 7.4, were heated at 90 °C for 10 min, ultrasonicated for 5 min, and then serially diluted to prepare solutions containing 2×10^2 to 1×10^6 cfu/mL of S. typhimurium and 3×10^2 to 1×10^6 of E. coli.

4. Biological functionalization of the chip

The chip-spotting layout is presented in Figure S1. Three out of the 10 MZIs were spotted with a 100 μg/mL S. typhimurium LPS solution, 4 MZIs with a 50 μg/mL E. coli LPS solution, and the remaining 3 with a 100 μg/mL BSA solution for the determination of non-specific binding.

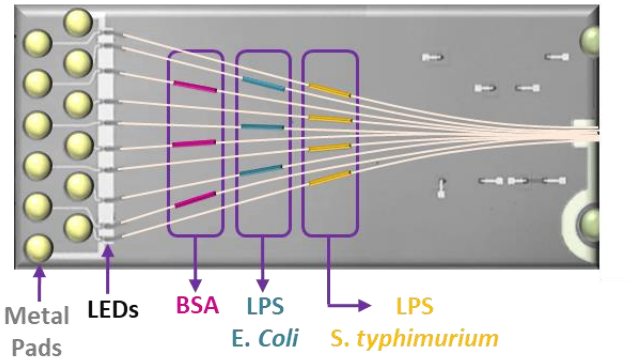

Figure S1. Depiction of chip-spotting layout.

5. Optimization of immobilized LPS concentration

The optimum concentration of bacteria LPS for immobilization onto the MZIs sensing window was determined by running sequentially over chips spotted with LPS solutions with concentrations ranging from 5 to 200 μg/mL, 1:1 (v/v) mixtures of zero calibrators with anti-bacteria antibodies (1 μg/mL), biotinylated anti-species-specific antibodies (10 μg/mL) and streptavidin (10 μg/mL) solutions, each one for 5 min, at a flow rate of 25 μL/min. As shown in Figure S2a, for E. *coli* the signal increased and reached a maximum value at LPS concentrations ranging between 50 and 100 μg/mL, whereas for higher concentrations the signal was slightly decreased. On the other hand, for S. *typhimurium* (Figure S2b) the maximum plateau signal values were obtained from chips spotted with LPS ≥ 100 μg/mL. Furthermore, for both bacteria, the sensor-to-sensor and the chip-to-chip signal variation was significantly improved (CV <5%) when the concentration of LPS used for coating was equal or higher than 50 μg/mL for both bacteria compared to the signal variations obtained from chips spotted with lower concentrations. Thus, the LPS concentration selected for spotting was 50 μg/mL and 100 μg/mL for S. *typhimurium* and E. *coli* LPS, respectively.

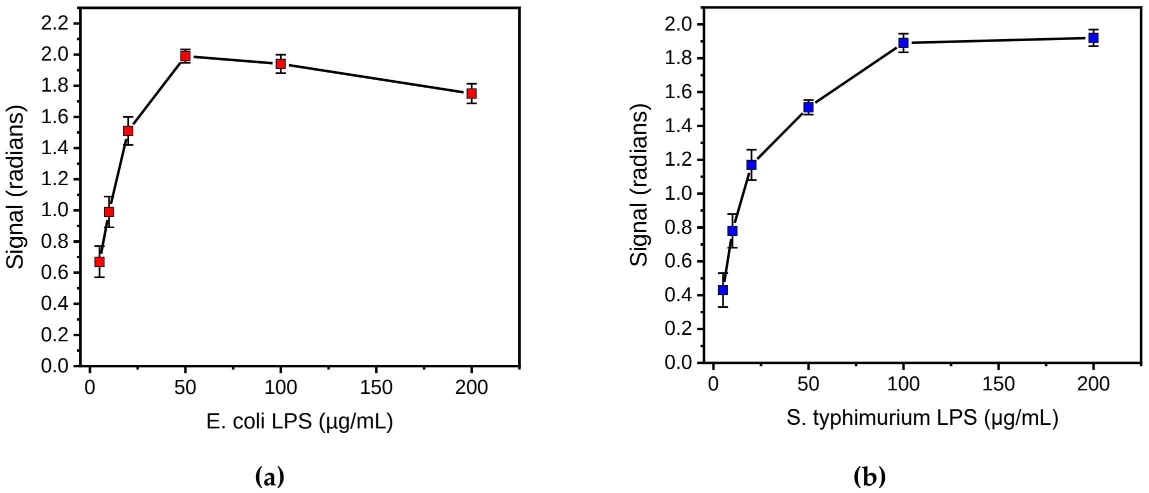

Figure S2. Zero calibrator signal values vs. the (**a**) E. *coli* and (**b**) S. *typhimurium* LPS concentration used for coating. Each point is the mean value of three measurements ± SD.

6. Optimization of Bacteria-Specific Antibodies Concentration

In Figure S3a and S3b, the signals obtained for a zero calibrator and a calibrator with LPS concentration of 250 ng/mL in mixtures with antibodies concentrations ranging from 0.25 to 2 μg/mL and from 0.375 to 3 μg/mL for E. *coli* and S. *typhimurium*, respectively, are

presented. As it is shown, for E. *coli* an adequate signal (≥ 1 rad) was achieved for anti-E. *coli* LPS concentrations ≥ 0.5 μg/mL. However, the higher inhibition using the 250 ng/mL calibrator was achieved for antibody concentration of 0.5 μg/mL. Similar results were observed for S. *typhimurium*, where the optimum antibody concentration was 0.75 μg/mL. Thus, these concentrations of bacteria-specific antibodies were selected for further experimentation.

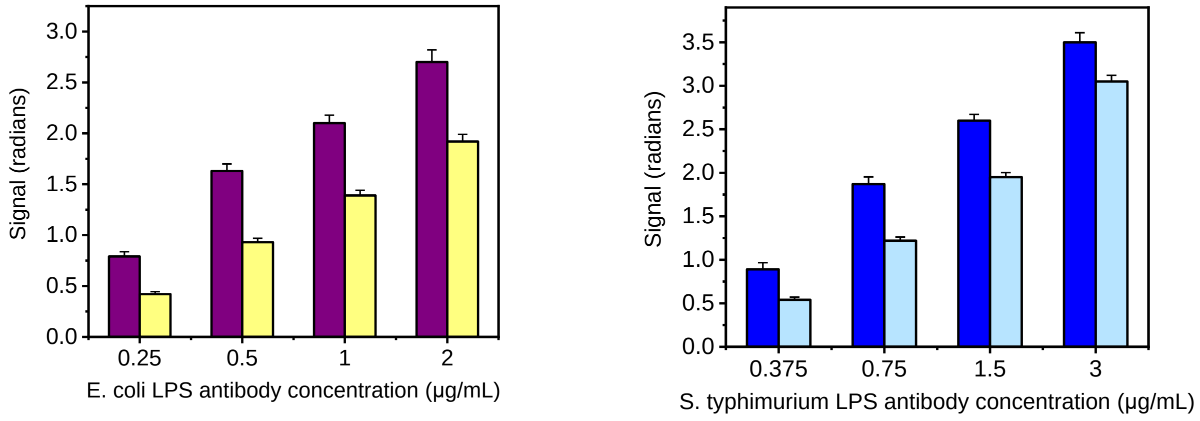

(a) **(b)**

Figure S3. (**a**) Signals corresponding to E. *coli* LPS zero calibrator (purple columns) or a calibrator with concentration 250 ng/mL (yellow columns) obtained for different concentrations of anti-E. *coli* antibody. (**b**) Signals corresponding to S. *typhimurium* LPS zero calibrator (dark blue columns) or a calibrator with concentration 250 ng/mL (light blue columns) obtained for different concentrations of anti-S. *typhimurium* antibody. Each point is the mean value of three measurements ± SD.

7. Optimization of Flow Rate/Assay Duration

The rate with which the reagents run over the chip determines the assay duration but also affects the assay performance. Thus, three different flow rates, 25, 35 and 50 μL/min, were tested to determine the highest possible one that did not affect the assay performance both in terms of absolute signal and detection sensitivity. It should be noticed that the flow rates tested resulted to a total assay duration (including the 3 steps of the immunoassay) of 12 min, 10 min and 6 min, respectively. In Figures S4a and S4b, the signal values obtained, applying the 3 different flow rates, for the zero calibrator, as well as a calibrator containing 250 ng/mL either E. *coli* or S. *typhimurium* LPS, are presented. As is indicated, by increasing the flow rate from 25 to 35 μL/min, the signal of zero calibrator was decreased by 15% for both bacteria, whereas a further increase of flow rate to 50 μL/min resulted in 55% signal loss compared to the signal obtained for the flow rate of 35 μL/min. In addition, the assay sensitivity was not affected when the flow rate was increased from 25 to 35. Thus, as a compromise between short assay duration, high absolute signal and detection sensitivity, the flow rate of 35 μL/min was adopted to the final protocol, resulting in total assay duration of only 10 min.

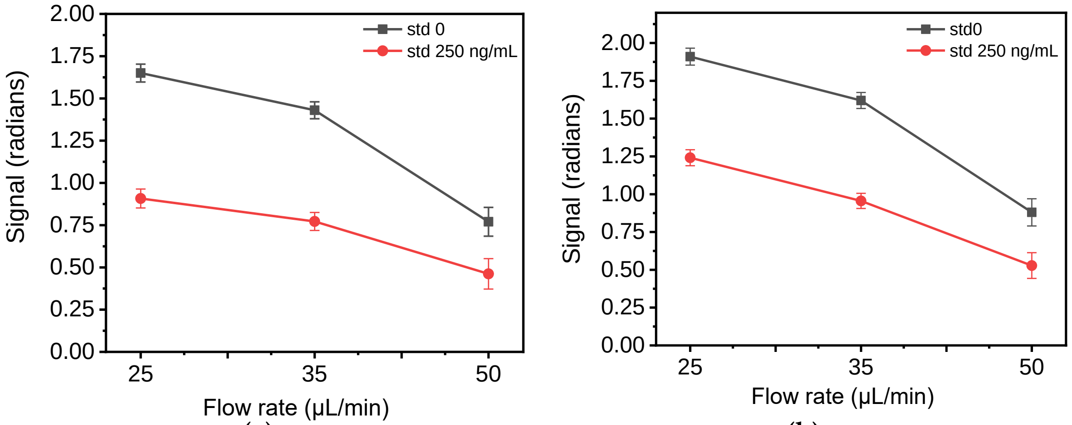

(a) **(b)**

Figure S4. Effect of flow rate on sensor responses obtained for a zero calibrator and a calibrator corresponding to 250 ng/mL for (**a**) E. *coli* and (**b**) S. *typhimurium* LPS. Each point is the mean value of three measurements ± SD.

8. Real-Time Responses from Dual-Analyte Chips

In Figure S5, real-time responses obtained from a chip on which different MZIs have been spotted with the LPS of E. *coli* (4 MZIs) or S. *typhimurium* (3 MZIs) for calibrators containing 1×10^3 to 1×10^6 cfu/mL bacteria, which have been subjected to heat-treatment and ultra-sonication, are presented.

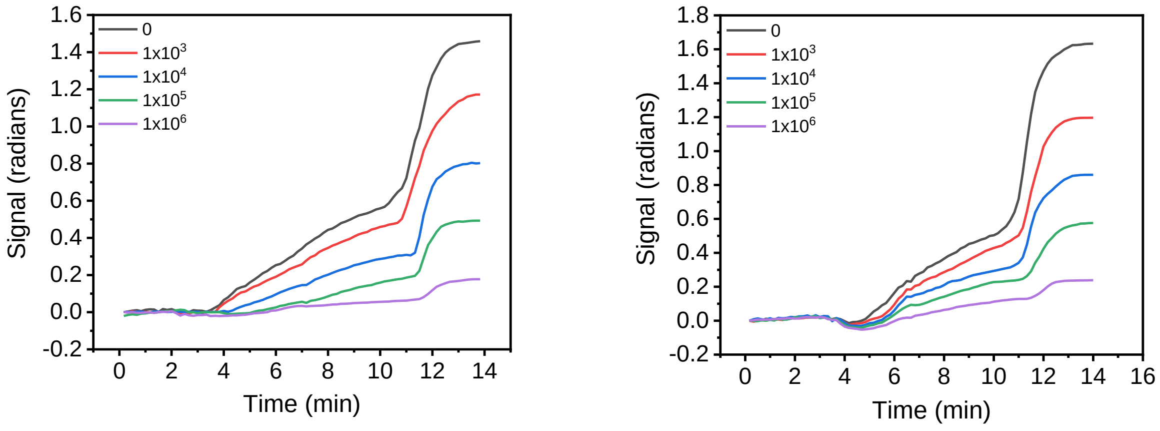

(a) **(b)**

Figure S5. Real-time responses provided from a chip on which different MZIs have been spotted with the LPS of (**a**) E. *coli* and (**b**) S. *typhimurium* upon running calibrators with concentrations ranging from 1×10^3 to 1×10^6 cfu/mL.

9. Recovery Experiments

Table S1. % Recovery values of S. *typhimurium*/E. *coli* bacteria spiked in two bottled water samples and two commercially available pasteurized milk samples.

**Sample**	**Amount Added (cfu/mL)**	**Amount Determined (cfu/mL)**		**% Recovery**	
		E. *coli*	S. *typhimurium*	E. *coli*	S. *tyhpimirium*
Bottled water (Avra)	5×10^3	4.9×10^3	5.1×10^3	99.0	102.5
	5×10^4	5.3×10^4	4.9×10^4	105	98.0

	5 × 10^5	4.7 × 10^5	4.5 × 10^5	94.0	90.0
Bottled water (Zagori)	5 × 10^3	4.6 × 10^3	4.8 × 10^3	92.0	96.0
	5 × 10^4	4.8 × 10^4	5.5 × 10^4	97.0	111
	5 × 10^5	5.1 × 10^5	5.1 × 10^5	101	102
Pasteurized milk (DELTA)	5 × 10^3	5.2 × 10^3	5.0 × 10^3	105	100
	5 × 10^4	5.6 × 10^4	4.7 × 10^4	112	94.0
	5 × 10^5	4.9 × 10^5	5.6 × 10^5	98.0	112
Pasteurized milk (NOUNOU)	5 × 10^3	5.5 × 10^3	5.5 × 10^3	110	109
	5 × 10^4	5.3 × 10^4	4.6 × 10^4	106	92.0
	5 × 10^5	4.6 × 10^5	5.5 × 10^5	92.0	110

10. Regeneration of the Immunosensor

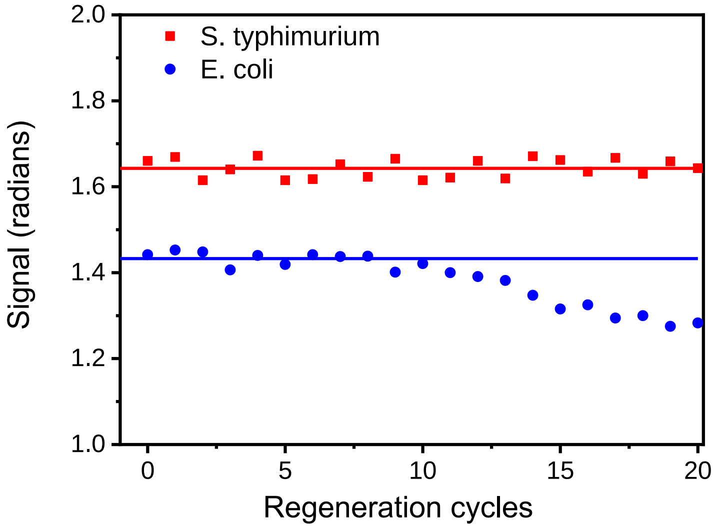

Figure S6. Signal obtained for the bacteria zero calibrator from a single chip after 20 repetitive regeneration/assay cycles. Horizontal red and blue lines correspond to the mean value of the 10 first measurements for S. *typhimurium* and E.*coli* assay, respectively.
